# The Mental Health Impact of the COVID-19 Pandemic: Implications for Sub-Saharan Africa

**DOI:** 10.2147/PRBM.S264286

**Published:** 2020-09-03

**Authors:** Bazghina-werq Semo, Souci Mogga Frissa

**Affiliations:** 1 Independent Global Health Consultant, Washington DC, DC, USA; 2 Department of Global Health, University of Washington, Seattle, WA, USA; 3 Institute of Psychiatry, Psychology, and Neuroscience, Centre for Global Mental Health, King’s College, London, UK

**Keywords:** COVID-19, mental health, sub-Saharan Africa

## Abstract

The COVID-19 pandemic is leading to mental health problems due to disease experience, physical distancing, stigma and discrimination, and job losses in many of the settings hardest hit by the pandemic. Health care workers, patients with COVID-19 and other illnesses, children, women, youth, and the elderly are experiencing post-traumatic stress disorders, anxiety, depression, and insomnia. Virtual mental health services have been established in many settings and social media is being used to impart mental health education and communication resources. This rapid review highlights mental health services across countries hardest hit by the COVID-19 pandemic. More needs to be done to take these services to scale and ensure equity and efficiency. The impact of COVID-19 on mental health in sub-Saharan Africa could be immense, given the weak health care systems. Similar to the Ebola epidemic of 2014–2016, COVID-19 is expected to cause anxiety, depression and post-traumatic stress disorders. Uptake of mental health care services is generally low, and communities rely on social resources. Hence, efforts to control the disease transmission should be contextualized. Low digital literacy, low smartphone penetration and limited internet connection make online mental health services a limited option for service delivery. Safeguarding social and cultural resilience factors and coping mechanisms is critical in the sub-Saharan African context. Mass media is a feasible way of providing social resources. Community health workers can be trained quickly to provide mental health education, screening and counselling services. Toll-free mental health helplines can be used to provide services to health care workers and those needing customized care. Mental health and psychosocial support services need to be integrated into the pandemic response and coordinated nationally. It is critical for these services to continue during and after the epidemic.

## Introduction

The COVID-19 (Coronavirus Disease of 2019) pandemic has impacted the lives of millions of people around the world and is likely to result in mental health problems among those with no previous mental illness as well as exacerbate the condition of those with pre-existing mental health problems/disorders. Mental health problems are likely to begin early and continue after the pandemic is over. Experience of the disease, breakdown of social support and stigma are possible causes of short-term mental health problems while factors such as economic losses can potentially cause long-term mental health issues.

## Aims and Methods

This paper summarizes the impact of COVID-19 on mental health in countries impacted by the pandemic and conceptualizes the implications for sub-Saharan Africa, with the aim of supporting time sensitive decisions that are required to impact policy and practice.

A rapid review of scientific articles and reports published in English before or on May 17, 2020 was conducted. For the first section of the manuscript, search words included COVID-19, AND mental health, SARS- COV-2 AND mental health, 2019-nCOV AND mental health. All scientific articles were obtained from the Medline database, while country and continent specific reports and preprint articles were abstracted using google^®^. A total of 21 scientific articles, 2 preprint articles and 6 country/continent specific reports and posts were included. For the second section which conceptualizes the implications for sub-Saharan Africa, the search words, Ebola AND mental health were used to explore all relevant journals in the Medline database. In total, 62 articles were screened, 3 manuscripts describing primary research and reviews from sub Saharan Africa were included. The rapid reviews were conducted by the two authors.

## Mental Health Impact of COVID-19

Several recently published articles have documented the mental health impact of the COVID-19 pandemic on different target populations along with available and acceptable modalities for preventing and treating these problems/disorders.

In the US 45% of adults have reported that they are experiencing anxiety and stress, these numbers are likely to increase as people continue to physically distance themselves as well as worry about getting sick from COVID-19.^1^ In the UK, approximately 33% of people are said to have experienced high levels of anxiety since the onset of the pandemic 2 while in Italy a survey identified posttraumatic stress symptoms (37%), stress (21%), severe anxiety (20%), depressive symptoms (17%) and insomnia (7%) among respondents.^3^

## Health Care and Other Frontline Workers

Settings in China, the US and elsewhere have reported that Health Care Workers (HCW), first responders and other frontline workers, experience significant clinical depression, anxiety, post-traumatic stress and suicidal ideation.^4^–^6^ In one study in China, the prevalence of depression among health care workers was 51%, anxiety 45%, insomnia 36% and 74% for post-traumatic stress symptoms.^4^ In China, there was no significant difference in mental health disorders between front line health workers caring for COVID-19 patients or other patients.^7^ In some settings, single doctors, nurses, and HCWs working in the emergency rooms have been found to have a greater predisposition to mental health problems.^8^,^9^ In Wuhan specifically, factors that were associated with mental health disorders among health workers included increased workload, lack of sleep, fear and discrimination.^10^ Additionally, a study conducted in Singapore and India demonstrated a strong association between physical symptoms such as headache, throat pain, lethargy etc., and mental health problems/disorders among health care workers.^11^

## COVID-19 Patients

A recent study conducted in China revealed 96.2% of COVID-19 inpatients experienced posttraumatic stress symptoms. These symptoms are likely to linger on for at least two years (as noted in studies conducted during the SARS outbreak) leading to reduced quality of life.^12^ Furthermore, some COVID-19 survivors, may develop long term consequences that require multidisciplinary care.^13^

## Children

The impact of the current COVID-19 pandemic on the mental health of children has been noted in Spain, where strict lockdown measures led to heightened levels of stress and anxiety among young children.^3^ Previous studies from pandemics have also shown children have the potential of developing adverse psychological conditions. Factors precipitating serious mental health conditions include quarantine away from parents/guardians and loss of a parent/s to illness. Additionally, children who are undergoing social distancing can potentially develop mental health issues precipitated by boredom and lack of social interaction with peers, though these may be less if children are sheltering with their families.^14^,^15^ Interestingly, a survey in China showed children under the age of 18 experienced low levels of stress probably due to the low rates of illness and death among this target group and the limited exposure to infection as a result of physical distancing.^16^

## Other Adults

Older adults are generally prone to mental health problems. In China approximately 23% of the older population (>55 years) have preexisting depression. Factors such as physical distancing and fear of death from COVID-19 are likely to result in stress and worsening of the mental health of seniors.^17^ In contrast, a recent survey on mental health conducted in the US and Canada revealed seniors (age 65 and above) experienced less mental health problems/disorders compared to other segments of the population. The reasons for this were not clear.^18^

The disruption in the health care system from COVID-19 is impacting the care of other patients with chronic illnesses who need to visit health care facilities for chronic care. It is believed, the high levels of stress due to the inability to access timely care can cause mental illness.^19^ In particular, psychiatric patients were noted to have higher rates of depression, anxiety and stress as well as intense suicidal ideation.^20^ Additionally, the disruption in the continued care of patients with pre-existing severe mental health conditions such as schizophrenia, and or bipolar disorder has the potential to cause worsening of both mental and physical health, since patients are highly reliant on regular clinical contacts for support.^21^

In a nationwide survey of psychological disorders among Chinese people during the COVID-19 pandemic, it was noted that women were affected more than men while the youth (age 18–30) had the highest COVID-19 peritraumatic distress index (CPDI).^16^ Similarly, in Canada and France, recent surveys showed mental health problems/disorders affected women more than men.^3^,^18^ The high levels of psychological disorders in women can be attributed to the fact that women are more vulnerable to the effects of traumatic events as well as to stress form the multiple roles they hold domestically and professionally.^3^,^16^,^22^,^23^

Similar to China, high levels of stress were noted among adolescents and youth in the US, Canada, and Belgium.^1^,^3^,^16^,^18^ These groups are at greatest risk for depression and suicidal ideation. In China, stress among the youth was likely to be caused by information coming from social media.^16^

Additionally, in China, stress levels were elevated among people with higher education levels due to increased self-awareness of their health, and migrant workers due to the uncertainty of income and exposure levels.^16^ Increased levels of stress, anxiety and depression were also noted among people with certain physical symptoms such as muscle aches, dizziness and coryza as well as in those who rated their health status as being poor.^24^ A good health care system with adequate preparedness and ongoing local information related to the pandemic was seen to lower stress levels among the general population.^16^ Other factors that were noted to be protective of mental health problems were high levels of trust in physicians, reduced risk of contracting COVID-19, adequate information about the disease and levels of personal precautionary measures.^25^ In the US, understandably, people earning lower wages reported higher levels of mental health problems compared to higher income people.^1^

Among the workforce returning to work in China, it was noted that prevention measures such as handwashing, face masks and measures at the workplace that improved workplace hygiene resulted in fewer mental health problems/disorders (post-traumatic stress disorders (PTSD), anxiety, depression, stress and insomnia) among the workforce.^26^

## Prevention and Treatment Modalities

To combat COVID-19 mental health problems, several settings have employed digital treatment and prevention solutions to achieve the desired scale. In particular virtual mental health services have worked well in addressing scale and limiting the exposure of patients to COVID-19 at health facilities.^18^,^27^ In China, mental health education and communication materials have been shared through commonly used social media platforms. Psychological counselling has been delivered for those in need of the service through applications such as WeChat.^4^ In some cases, artificial intelligence has been used to identify posts on social media from people who are in crisis and likely to commit suicide. Those at risk are then reached proactively.^4^ In Singapore, online psychotherapy is being provided through videoconferencing, while cognitive behavioral and mindfulness-based therapies are provided through smartphone applications.^9^ In Canada, employers are providing mental health support to their employees by waiving fees for mental health services, offering additional support and increasing awareness of mental health among their employees. Virtual care for mental health has been established and 62% of Canadians seeking services are satisfied with the level of service.^18^ In the US, federal hotlines and online therapy services that predate the COVID-19 pandemic are being utilized more and more by emotionally distressed people leading to a marked increase in the use of the services.^5^

## Lessons Learned in Service Delivery

In China, several concerns regarding the current state of mental health services have been documented. While several services have been initiated to combat and address mental health issues, efficiency and equity in service provision may be lacking due to insufficient national coordination. Additionally, the efficacy of these services is not being evaluated, leaving behind the opportunity for continuous quality improvement. Online mental health services are helpful for those who have the resources and literacy to access them but the lack of alternative services is leaving behind a population that does not have access to the technology or literacy required to receive these services.^19^,^28^

Another challenge that has been noted with regard to services in some settings is the lack of collaboration between health care facilities providing mental health services and community centers. Continuity of care for patients becomes a challenge once they are discharged from health care facilities. Inadequate numbers of mental health professionals such as psychologists and psychiatrists have led to front line health workers taking on the provision of mental health services. In China, the lack of policy and standardized training in mental health for non-mental health professionals may affect the quality of service provision.^19^

In the US, existing mental health services are not sufficient to deal with the demand for services. Some of the challenges include a restriction on licensing and reimbursement of therapists who are trying to set up virtual services and the closure of community behavioral centers that are struggling to remain solvent.^5^

Lessons learned from some of the countries with established services include the following: the strengthening of mental health services during the COVID-19 pandemic requires identifying high-risk groups early on and providing them with targeted interventions. An important feature of this is to conduct routine screening of mental health problems. Due to limited mental health care staff in many settings, this can be conducted by other front-line health workers. Additionally, the mental health of front-line health workers can be preserved by ensuring shorter workdays, provision of protective gear and adequate training in infection control. Stigma and discrimination of COVID-19 patients and front-line health workers need to be addressed by national authorities and influential leaders who should also take on the responsibility of ensuring the public receive accurate messaging during the course of the pandemic.^9^

## Implications for Sub-Saharan Africa

To date, very little has been documented about the impact of COVID-19 on mental health in sub-Saharan Africa. A global analysis of the literature on COVID-19 highlights the need for research from Africa which remains under researched.^29^

In recent news the WHO warned that in the first year of the pandemic, Africa could see as many as 44 million people infected with COVID-19 and estimates up to 190 000 deaths among Africans from COVID-19 depending on the intervention measures taken to stop the spread.^30^ If these estimates are close to accurate, we predict the impact on mental health will be immense due to sub-Saharan Africa’s weak health care systems, unless social and cultural resilience factors and coping mechanisms are safeguarded. Causes for mental health problems/disorders will remain similar to ones observed/documented in other settings. We will discuss these in greater detail below.

## Similar Mental Health Consequences in Both Ebola and COVID-19

To better understand the impact of COVID-19 on mental health, we examined the literature on the recent Ebola epidemic in sub-Saharan Africa. The 2014–2016 West Africa Ebola virus outbreak triggered serious psychosocial consequences at both individual and community levels. Stress, grief, anxiety, depression and symptoms of PTSD were reported at the individual level while stigma, discrimination and interruption of social networks were observed at the community level.^31^

In addition to some of the well-documented predictors of mental health problems, the inability of families to care for sick relatives coupled with the inability of family members to perform traditional and religious burial rituals for loved ones caused psychological distress.^32^ More importantly, extreme fear/anxiety and fear-related behavior during the epidemic formed the most difficult barrier to preventive and curative measures and led to a crisis.^33^

The mental health consequences of the Ebola outbreak persisted after the end of the epidemic. In a survey carried out in Sierra Leone among a nationally representative sample of the population, 48% of participants reported at least one symptom of anxiety or depression and 76% reported PTSD symptoms a year into the epidemic.^34^ Symptoms of mental health disorders were common especially among those with Ebola related experiences including those who knew someone who had died from Ebola or been quarantined due to Ebola exposure.^34^ Survivors were distressed by traumatic memories and the rejection by society, while those who never contracted the disease grieved for lost relatives or struggled to cope with extreme anxiety.^31^

Although Ebola is deadlier and less contagious than COVID-19, we think the mental health consequences arising from the COVID-19 pandemic in sub-Saharan Africa will have some similarity to the West Africa Ebola outbreak.

Since March 2020, countries in Africa have implemented physical distancing measures, to reduce transmission of COVID-19, mainly closing educational institutions and banning mass gatherings. Twenty-two countries have also implemented a lockdown; 12 of which applied a national lockdown while the rest applied lockdown measures in affected areas.^35^

## Factors Facilitating Mental Health Problems/Disorders

Three major factors are impacting the population and may lead to mental health problems:

1. The direct impact of the disease, particularly near-death experience during illness, isolation from loved ones during hospitalization which is a grim experience both for the family and the patients in most African communities, stress from news about high death rates among the very ill and the highly exposed (eg health care workers), loss of loved ones or parents/guardians and stigma and discrimination among survivors and affected families. In particular, mental health problems will disproportionately affect frontline health care workers, COVID-19 survivors, families and children who have lost loved ones to the disease and those with pre-existing mental disorders
2. Efforts to contain the spread of COVID-19 through restricting and limiting physical interactions may lead to limited access to social support structures, inadequate supply of food and medication, limited access to treatment for those with existing mental health problems as well as other chronic conditions, restricted access to faith-based institutions and leaders due to the ban on social gatherings, lack of access and support for those suffering from intimate partner violence or other forms of abuse. Most Sub-Saharan African communities are structured traditionally with a strong community and neighborhood focus and the main source of support and care for older people is family. Hence, in most communities, older people are protected from the calamities observed in care homes in high-income countries. However, the physical distancing directives might lead to loneliness in some settings.
3. Uncertainty and stress resulting from loss of jobs and livelihoods.

The stress of losing jobs and livelihoods can be overwhelming for many but this is worse for people of low socioeconomic status including those working in the informal sector. Although several countries have put some measures in place to mitigate the economic difficulties, the financial packages have been minimal, while those working in informal businesses have not been eligible for any of the government schemes.^36^

The lockdown and the physical distancing measures are being debated and questioned by many about their impact on the informal economy which provides a livelihood for 60% of men, and nearly 75% of women and the majority of city dwellers who rely on this economy for their day to day needs.^37^,^38^ Additionally, farmers are being affected badly by lockdown measures as their perishable products are not reaching the cities as expected. Executing lockdown and physical distancing measures without sufficient mitigating measures to help informal businesses and individuals cope economically will lead to uncertainty, loss of livelihood and financial strain which will have a negative impact on mental health. In Sub Saharan Africa “Communal living is not just about culture, it is a matter of economic survival”.^39^

## Preventing and Addressing Mental Health Problems/Disorders in Sub-Saharan Africa

Mental health services will need to be prioritized during the COVID-19 pandemic in sub-Saharan Africa. However, improving access and utilization of these needs to be thought through given some of the contextual factors. The utilization of mental health services does not happen frequently because many people in sub-Saharan Africa only engage with the health care system for services when all other social resources and self-help methods fail or if symptoms get very severe.^40^ Some of the resources people access for relief from stress and mental problems include keeping in touch with others, attending faith and religious events, engaging in prayers and reading scriptures.^41^–^52^ The COVID-19 lockdowns in sub-Saharan Africa have hindered access to social resources.

Online and digital platforms used in high income settings are a limited option for mental health education and counselling services in sub-Saharan Africa mostly due to low smartphone penetration and internet access. The internet usage gap varies among countries (6.3% Chad-87% in Kenya).^53^ Additionally, digital literacy is a limiting factor for many and likely worse than noted in settings like China. Those who will benefit from online and digital platforms will likely be populations living in urban areas.

In most African countries, ministries of health and other responsible government institutions conduct regular press releases on COVID-19 and disseminate key information to the public, working in partnership with local radio and television stations. To make the services and social resources that are lacking during the lockdown accessible, we recommend employing mass media for communication of self-help measures that are likely to reduce stress. Mass media can also be used to communicate survivor experiences. Although there are few examples of people discussing their recovery experience, (eg Oluwaseun Osowobi on BBC and testimonies of patients in Burkina Faso) a lot more can be done to prepare and help others cope better as well as provide education on stigma and discrimination.^54^ TV and radio should be used frequently to broadcast religious services and relevant talk shows that can help improve mental health.

We foresee greater challenges in the provision of mental health services compared to those documented in other settings. Lack of national coordination, shortage of mental health care staff etc., are issues that need to be addressed. Guidelines available for emergency situations^55^ can be adapted for use in COVID-19 addressing the cultural and organizational context and taking in to account the responses needed to contain the spread of the disease. The role of mental health care professionals including psychiatrists, psychologists and mental health nurses is to provide mental health leadership and to guide the development of an intersectoral approach, while looking after, protecting and supporting their existing caseloads.

Many sub-Saharan African countries rely heavily on community health workers (CHW)/village health workers to deliver services.^56^ With appropriate personal protection measures, this cadre of workers can be quickly trained to provide case detection, one on one counselling, psychosocial screening and support, stigma reduction, and community-based rehabilitation messaging within communities.^57^ In countries where community support groups exist for other diseases, these may be utilized to provide aspects of the above.

To augment this, toll-free mental health helplines available in some countries in Sub-Saharan Africa can be scaled up inexpensively to provide more in-depth and anonymous counselling for select segments of the population such as health care workers and people experiencing suicidal ideation. Several tiers of helplines can be established depending on the complexity of cases and the need for services.

In conclusion, addressing the mental health impact of COVID-19 in sub-Saharan Africa needs to be prioritized. National coordination in service provision is key. Both mental health and psychosocial support need to be integrated into the pandemic response. Treatment efforts should be coupled with a response to support the psychosocial needs of patients and their families. Training of facility and community-based health workers needs to be coordinated while guidelines for mental health support during outbreaks need to be made available and disseminated to all providing services. Lockdown and physical distancing measures should be contextualized while cultural attributes and resources that provide social support and resilience should be tapped. There is a critical need for strong communication and community engagement to combat misinformation and fear-related behaviors. Psychosocial support including other interventions must continue during and after the pandemic targeting high-risk groups like survivors, bereaved family members, children who lost their parents, health care workers, burial workers and other first-line responders.

## References

[1] Panchal N, Kamal R, Orgera K, Cox C, Garfield R, Hamel L, Muñana C, and Chidambaram P. The Implications of COVID-19 for Mental Health and Substance Use. Available from:https://www.kff.org/coronavirus-covid-19/issue-brief/the-implications-of-covid-19-for-mental-health-and-substance-use/. Accessed 5 7, 2020.

[2] Office for National Statistics, Statistical bulletin. Coronavirus and the latest indicators for the UK economy and society; 5 14, 2020.

[3] United Nations Regional Information Center for Europe. Concerns are raised over the threat COVID-19 to mental health in Europe. Available from https://unric.org/en/concerns-are-raised-over-the-threat-of-covid-19-to-mental-health-in-europe/. Accessed 5 7, 2020.

[4] Liu S, Yang L, Zhang C, et al. Online mental health services in China during the COVID-19 outbreak. *Lancet Psychiatry*. 2020;7(4):e17–e18. doi:10.1016/S2215-0366(20)30077-8 32085841 PMC7129099

[5] Wan W. The Coronavirus pandemic is pushing America into a mental health crisis. Available from www.washingtonpost.com/health/2020/05/04/mental-health-coronavirus/. Accessed 5 4, 2020.

[6] Rossi R, Socci V, Pacitti F, et al. Mental health outcomes among front and second line health workers associated with the COVID-19 pandemic in Italy. 2020;1-5. doi:10.1101/2020.04.16.20067801 PMC7256664 32463467

[7] Liang Y, Chen M, Zheng X, Liu J. Screening for Chinese medical staff mental health by SDS and SAS during the outbreak of COVID-19. *J Psychosom Res*. 2020;133:110102. doi:10.1016/j.jpsychores.2020.110102 32224344 PMC7139244

[8] Lai J, Ma S, Wang Y, et al. Factors associated with mental health outcomes among health care workers exposed to coronavirus disease 2019. *JAMA Netw Open*. 2020;3(3):e203976. doi:10.1001/jamanetworkopen.2020.3976 32202646 PMC7090843

[9] Ho C, Chee C, Ho R. Mental health strategies to combat the psychological impact of coronavirus disease 2019 (COVID-19) beyond paranoia and panic. *Ann Acad Med Singapore*. 2020;49:1–3. 32200399

[10] Chen Q, Liang M, Li Y, et al. Mental health care for medical staff in China during the COVID-19 outbreak [published correction appears in Lancet Psychiatry. 2020 May;7(5):e27]. *Lancet Psychiatry*. 2020;7(4):e15–e16. doi:10.1016/S2215-0366(20)30078-X 32085839 PMC7129426

[11] Chew NWS, Lee GKH, Tan BYQ, et al. A multinational, multicentre study on the psychological outcomes and associated physical symptoms amongst healthcare workers during COVID-19 outbreak [published online ahead of print, 2020 Apr 21]. *Brain Behav Immunol*. 2020. doi:10.1016/j.bbi.2020.04.049 PMC7172854 32330593

[12] Bo HX, Li W, Yang Y, et al. Posttraumatic stress symptoms and attitude toward crisis mental health services among clinically stable patients with COVID-19 in China [published online ahead of print, 2020 Mar 27]. *Psychol Med*. 2020:1–2. doi:10.1017/S0033291720000999. PMC7200846 32216863

[13] Gemelli Against COVID-19 Post-Acute Care Study Group. Post-COVID-19 global health strategies: the need for an interdisciplinary approach [published online ahead of print, 2020 Jun 11]. *Aging Clin Exp Res*. 2020:1–8. doi:10.1007/s40520-020-01616-x 32529595 PMC7287410

[14] Wang G, Zhang Y, Zhao J, Zhang J, Jiang F. Mitigate the effects of home confinement on children during the COVID-19 outbreak. *Lancet*. 2020;395(10228):945–947. doi:10.1016/S0140-6736(20)30547-X PMC7124694 32145186

[15] Liu JJ, Bao Y, Huang X, Shi J, Lu L. Mental health considerations for children quarantined because of COVID-19. *Lancet Child Adolesc Health*. 2020;4(5):347–349. doi:10.1016/S2352-4642(20)30096-1 32224303 PMC7118598

[16] Qiu J, Shen B, Zhao M, Wang Z, Xie B, Xu Y. A nationwide survey of psychological distress among Chinese people in the COVID-19 epidemic: implications and policy recommendations [published correction appears in Gen Psychiatr. 2020 Apr 27;33(2):e100213corr1]. *Gen Psychiatry*. 2020;33(2):e100213. doi:10.1136/gpsych-2020-100213 PMC7061893 32215365

[17] Yang Y, Li W, Zhang Q, Zhang L, Cheung T, Xiang YT. Mental health services for older adults in China during the COVID-19 outbreak. *Lancet Psychiatry*. 2020;7(4):e19. doi:10.1016/S2215-0366(20)30079-1 32085843 PMC7128970

[18] BioSpace. New Study by Teladoc Health Reveals COVID-19 Pandemic’s Widespread Negative Impact on Mental Health. May 06, 2020. Available from: https://www.biospace.com/article/releases/new-study 560 -by-teladoc-health-reveals-covid-19-pandemic-s-widespread-negative-impact-on-mental-health/. Accessed May 6, 2020.

[19] Duan L, Zhu G. Psychological interventions for people affected by the COVID-19 epidemic. *Lancet Psychiatry*. 2020;7(4):300–302. doi:10.1016/S2215-0366(20)30073-0 32085840 PMC7128328

[20] Hao F, Tan W, Jiang L, et al. Do psychiatric patients experience more psychiatric symptoms during COVID-19 pandemic and lockdown? A case-control study with service and research implications for immunopsychiatry. *Brain Behav Immunol*. 2020;87:100–106. doi:10.1016/j.bbi.2020.04.069 PMC7184991 32353518

[21] World Health Organization. Meeting report on excess mortality in person with severe mental disorder; 18–20 November 2015. WHO, Geneva; 2016 Available from: https://www.who.int/mental_health/evidence/excess_mortality_meeting_report.pdf?ua=1. Accessed 712, 2020.

[22] Breslau N, Kessler RC, Chilcoat HD, Schultz LR, Davis GC, Andreski P. Trauma and posttraumatic stress disorder in the community: the 1996 detroit area survey of trauma. *Arch Gen Psychiatry*. 1998;55(7):626–632. doi:10.1001/archpsyc.55.7.626 9672053

[23] Breslau N. The epidemiology of posttraumatic stress disorder: what is the extent of the problem? *J Clin Psychiatry*. 2001;62(Suppl 17):16–22. 11495091

[24] Wang C, Pan R, Wan X, et al. Immediate psychological responses and associated factors during the initial stage of the 2019 Coronavirus Disease (COVID-19) epidemic among the general population in China. *Int J Environ Res Public Health*. 2020;17(5):1729. doi:10.3390/ijerph17051729 PMC7084952 32155789

[25] Wang C, Pan R, Wan X, et al. A longitudinal study on the mental health of general population during the COVID-19 epidemic in China. *Brain Behav Immunol*. 2020;87:40–48. doi:10.1016/j.bbi.2020.04.028 PMC7153528 32298802

[26] Tan W, Hao F, McIntyre RS, et al. Is returning to work during the COVID-19 pandemic stressful? A study on immediate mental health status and psychoneuroimmunity prevention measures of Chinese workforce. *Brain Behav Immunol*. 2020;87:84–92. doi:10.1016/j.bbi.2020.04.055 PMC7179503 32335200

[27] Zhou X, Snoswell CL, Harding LE, et al. The role of telehealth in reducing the mental health burden from COVID-19. *Telemed J E Health*. 2020;26(4):377–379. doi:10.1089/tmj.2020.0068 32202977

[28] Torous J, Jän Myrick K, Rauseo-Ricupero N, Firth J. Digital mental health and COVID-19: smorrow. *JMIR Ment Health*. 2020;7(3):e18848. doi:10.2196/18848 32213476 PMC7101061

[29] Tran BX, Ha GH, Nguyen LH, et al. Studies of novel coronavirus disease 19 (COVID-19) pandemic: a global analysis of literature. *Int J Environ Res Public Health*. 2020;17:4095. doi:10.3390/ijerph17114095 PMC7312200 32521776

[30] WHO regional office for Africa, May 7 2020. New WHO estimates: up to 190 000 people could die of COVID-19 in Africa if not controlled; 5 7, 2020 Available from: https://www.afro.who.int/news/new-who-estimates-190-000-people-could-die-covid-19-africa-if-not-controlled. Accessed 72, 2020.

[31] Shultz JM, Cooper JL, Baingana F, et al. The role of fear-related behaviours in the 2013–2016 West Africa ebola virus disease outbreak. *Curr Psychiatry Rep*. 2016;18:104. doi:10.1007/s11920-016-0741-y 27739026 PMC5241909

[32] O’Leary A, Jalloh MF, Neria Y. Fear and culture: contextualising mental health impact of the 2014–2016 Ebola epidemic in West Africa. *BMJ Glob Health*. 2018;3:e000924. doi:10.1136/bmjgh-2018-000924 PMC6035506 29989048

[33] Chan M. ebola virus disease in west africa — no early end to the outbreak. *N Engl J Med*. 2014;371:1183–1185. doi:10.1056/NEJMp1409859 25140856

[34] Jalloh MF, Li W, Bunnell RE, et al. Impact of Ebola experiences and risk perceptions on mental health in Sierra Leone, July 2015. *BMJ Glob Health*. 2018;3:e000471. doi:10.1136/bmjgh-2017-000471 PMC5873549 29607096

[35] WHO regional office for Africa. May 5 2020. COVID-19 WHO African Region External situation report 10; 5 5, 2020 Available from: https://apps.who.int/iris/bitstream/handle/10665/331989/SITREP_COVID-19_WHOAFRO_20200506-eng.pdf. Accessed 425, 2020.

[36] International Monitory Fund. Policy responses to COVID-19. Available from: https://www.imf.org/en/Topics/imf-and-covid19/Policy-Responses-to-COVID-19. Accessed 425, 2020.

[37] Lashitew A, Social distancing unlikely to hold up in Africa without a safety net for microentrepreneurs. AFRICA IN FOCUS; 4 9, 2020 Available from: https://www.brookings.edu/blog/africa-in-focus/2020/04/09/social-distancing-unlikely-to-hold-up-in-africa-without-a-safety-net-for-microentrepreneurs. Accessed 425, 2020.

[38] United Nations Economic Commission for Africa. COVID-19 in Africa: protecting lives and economies. Available from: https://www.uneca.org/publications/covid-19-africa-protecting-lives-and-economies. Accessed 425, 2020.

[39] Soludo CC COVID-19: can Africa Afford Lockdowns? By Chukwuma Charles Soludo Premium Times; 4 24, 2020 Available from: https://opinion.premiumtimesng.com/2020/04/24/covid-19-can-africa-afford-lockdowns-by-chukwuma-charles-soludo. Accessed 425, 2020.

[40] Mayston R, Frissa S, Tekola B, Hanlon C, Prince M, Fekadu A. Explanatory models of depression in sub-Saharan Africa: synthesis of qualitative evidence. *Soc Sci Med*. 2020;246. doi:10.1016/j.socscimed.2019.112760. PMC7014569 32006814

[41] Patel V, Gwanzura F, Simunyu E, Lloyd K, Mann A. The phenomenology and explanatory models of common mental disorder: a study in primary care in Harare, Zimbabwe. *Psychol Med*. 1995a;25(6):1191–1199. doi:10.1017/S003329170003316X 8637949

[42] Patel V, Musara T, Butau T, Maramba P, Fuyane S. Concepts of mental illness and medical pluralism in Harare. *Psychol Med*. 1995b;25(3):485–493. doi:10.1017/S0033291700033407 7480429

[43] Okello ES, Ekblad S. Lay concepts of depression among the Baganda of Uganda: a pilot study. *Transcult Psychiatry*. 2006;43(2):287–313. doi:10.1177/1363461506064871 16893877

[44] Familiar I, Sharma S, Ndayisaba H, Munyentwari N, Sibomana S, Bass JK. Community perceptions of mental distress in a post-conflict setting: a qualitative study in Burundi. *Glob Public Health*. 2013;8(8):943–957. doi:10.1080/17441692.2013.819587 23941217

[45] Petersen I, Hancock JH, Bhana A, Govender K; Mental Health Care (PRIME). Closing the treatment gap for depression co-morbid with HIV in South Africa: voices of afflicted women. *Health*. 2013;5(3A):557–566. doi:10.4236/health.2013.53A074

[46] Monteiro N, Balogun SK. Perceptions of mental illness in Ethiopia: a profile of attitudes, beliefs and practices among community members, healthcare workers and traditional healers. *Int J Cult Ment Health*. 2014;7(3):259–272. doi:10.1080/17542863.2013.784344

[47] Nakimuli-Mpungu E, Wamala K, Okello J, et al. Developing a culturally sensitive group support intervention for depression among HIV infected and non-infected Ugandan adults: a qualitative study. *J Affect Disord*. 2014;163:10–17. doi:10.1016/j.jad.2014.03.042 24836082

[48] Irankunda P, Heatherington L. Mental health treatment outcome expectancies in Burundi. *Transcult Psychiatry*. 2017;54(1):46–65. doi:10.1177/1363461516652302 27317669

[49] Johnson LR, Chin EG, Kajumba M, Buchanan E, Kizito S, Bangirana P. Doconcepts of depression predict treatment pathways? A closer look at explanatory models among clinical and nonclinical samples in Uganda. *J Clin Psychol*. 2017a;73(7):893–909. doi:10.1002/jclp.22378 27805737

[50] Johnson LR, Chin EG, Kajumba M, Kizito S, Bangirana P. Views on depression From Traditional healing and psychiatry clinics in Uganda: perspectives from patients and their providers. *J Cross Cult Psychol*. 2017b;48(2):243–261. doi:10.1177%2F0022022116675424

[51] Murray SM, Familiar I, Nakasujja N, et al. Caregiver mental health and HIV-infected child wellness: perspectives from Ugandan caregivers. *AIDS Care*. 2017;29(6):793–799. doi:10.1080/09540121.2016.1263722 27951734 PMC5555603

[52] Myers B, Joska JA, Lund C, et al. Patient preferences for the integration of mental health counseling and chronic disease care in South Africa. *Patient Prefer Adherence*. 2018;12:1797–1803. doi:10.2147/PPA.S176356 30271123 PMC6154740

[53] Internet World Stats. Usage and Population Statistics. Available from: https://www.internetworldstats.com/stats1.htm. Accessed 425, 2020.

[54] BBC News. Coronavirus in Nigeria: activist Oluwaseun Osowobi on recovering from Covid-19. Available from: https://www.bbc.co.uk/news/av/world-africa-52257050/coronavirus-in-nigeria-activist-oluwaseun-osowobi-on-recovering-from-covid-19. Accessed 87, 2020.

[55] Inter-Agency Standing Committee (IASC). *IASC Guidelines on Mental Health and Psychosocial Support in Emergency Settings*. Geneva: IASC; 2007. 10.1080/09540261.2022.2147420 36502397

[56] WHO. Strengthening the performance of community health workers in primary health care. Report of a WHO Study Group. Geneva, World Health Organization (WHO Technical Report Series, No. 780); 1989. 2497585

[57] Fekadu A, Hanlon C, Medhin G, et al. Development of a scalable mental healthcare plan for a rural district in Ethiopia. *Br J Psychiatry*. 2016;208:s4–s12. doi:10.1192/bjp.bp.114.153676 26447174 PMC4698551

